# Estimates of alcohol-related oesophageal cancer burden in Japan: systematic review and meta-analyses

**DOI:** 10.2471/BLT.14.142141

**Published:** 2015-02-24

**Authors:** Michael Roerecke, Kevin D Shield, Susumu Higuchi, Atsushi Yoshimura, Elisabeth Larsen, Maximilien X Rehm, Jürgen Rehm

**Affiliations:** a Centre for Addiction and Mental Health (CAMH), Social and Epidemiological Research Department, Room T523, 33 Russell Street, Toronto, Ontario, M5S 2S1, Canada.; b National Hospital Organization, Kurihama Medical and Addiction Center, Yokosuka, Japan.; c Ryerson University, Toronto, Canada.

## Abstract

**Objective:**

To refine estimates of the burden of alcohol-related oesophageal cancer in Japan.

**Methods:**

We searched PubMed for published reviews and original studies on alcohol intake, aldehyde dehydrogenase polymorphisms, and risk for oesophageal cancer in Japan, published before 2014. We conducted random-effects meta-analyses, including subgroup analyses by aldehyde dehydrogenase variants. We estimated deaths and loss of disability-adjusted life years (DALYs) from oesophageal cancer using exposure distributions for alcohol based on age, sex and relative risks per unit of exposure.

**Findings:**

We identified 14 relevant studies. Three cohort studies and four case-control studies had dose–response data. Evidence from cohort studies showed that people who consumed the equivalent of 100 g/day of pure alcohol had an 11.71 fold, (95% confidence interval, CI: 2.67–51.32) risk of oesophageal cancer compared to those who never consumed alcohol. Evidence from case-control studies showed that the increase in risk was 33.11 fold (95% CI: 8.15–134.43) in the population at large. The difference by study design is explained by the 159 fold (95% CI: 27.2–938.2) risk among those with an inactive aldehyde dehydrogenase enzyme variant. Applying these dose–response estimates to the national profile of alcohol intake yielded 5279 oesophageal cancer deaths and 102 988 DALYs lost – almost double the estimates produced by the most recent global burden of disease exercise.

**Conclusion:**

Use of global dose–response data results in an underestimate of the burden of disease from oesophageal cancer in Japan. Where possible, national burden of disease studies should use results from the population concerned.

## Résumé

**Objectif:**

Affiner les estimations de la charge du cancer de l'œsophage lié à l'alcool au Japon.

**Méthodes:**

Nous avons effectué une recherche bibliographique dans PubMed pour trouver les revues publiées et les études originales sur la consommation d'alcool, les polymorphismes de l'aldéhyde déshydrogénase et le risque de développement d'un cancer de l'œsophage au Japon, publiées avant 2014. Nous avons réalisé des méta-analyses à effet aléatoire, y compris des analyses de sous-groupe par variant de l'aldéhyde déshydrogénase. Nous avons estimé les décès et l'espérance de vie corrigée de l'incapacité (EVCI) dus au cancer de l'œsophage en utilisant les distributions de l'exposition à l'alcool en fonction de l'âge, du sexe et des risques relatifs par unité d'exposition.

**Résultats:**

Nous avons identifié 14 études pertinentes. Trois études de cohortes et quatre études de cas-témoins présentaient des données de dose-réponse. Les données issues des études de cohortes montrent que les personnes ayant consommé l'équivalent de 100 g/jour d'alcool pur avaient un risque 11,71 fois supérieur (intervalle de confiance à 95% [IC]: 2,67–51,32) de développer un cancer de l'œsophage par rapport aux personnes qui n'ont jamais consommé d'alcool. Les données obtenues à partir des études de cas-témoins ont montré que l'augmentation du risque était 33,11 fois supérieure (IC à 95%: 8,15–134,43) par rapport à la population en général. La différence par la conception de l'étude est expliquée par un risque 159 fois supérieur (IC à 95%: 27,2–938,2) parmi les personnes présentant un variant enzymatique inactif de l'aldéhyde déshydrogénase. En appliquant ces estimations de dose-réponse au profil national de consommation d'alcool, nous sommes arrivés à 5279 décès et 102 988 EVCI dus au cancer de l'œsophage – soit presque le double des estimations produites par la plus récente charge globale de la maladie.

**Conclusion:**

L'utilisation de données globales de dose-réponse se traduit par une sous-estimation de la charge de morbidité pour le cancer de l'œsophage au Japon. Dans la mesure du possible, la charge nationale des études portant sur les maladies devrait utiliser les résultats obtenus sur la population concernée.

## Resumen

**Objetivo:**

Refinar las estimaciones de la carga del cáncer esofágico relacionado con el consumo de alcohol en Japón.

**Métodos:**

Se buscaron revisiones y estudios originales publicados antes de 2014 sobre la ingesta de alcohol, polimorfismos del aldehído deshidrogenasa y el riesgo de cáncer de esófago en Japón en la base de datos PubMed. Se efectuaron metaanálisis de efectos aleatorios, que incluían análisis de subgrupos de variantes del aldehído deshidrogenasa y se estimaron las muertes y la pérdida de años de vida ajustados por discapacidad (AVAD) por cáncer de esófago mediante distribuciones de exposición para el alcohol basadas en la edad, el sexo y los riesgos relativos por unidad de exposición.

**Resultados:**

Se identificaron 14 estudios pertinentes. Tres estudios de cohorte y cuatro estudios de casos y controles contenían datos sobre la respuesta en relación con la dosis. Las pruebas de los estudios de cohorte demostraron que el riesgo de cáncer esofágico de que quienes consumen el equivalente a 100 g/día de alcohol puro era 11,71 veces mayor (intervalo de confianza del 95 % [IC]: 2,67–51,32) en comparación con aquellos que nunca consumieron alcohol. Las pruebas de los estudios de casos y controles demostraron que el riesgo aumentó 33,11 veces (IC del 95 %: 8,15–134,43) en la población en general. La diferencia por el diseño del estudio se explica por el aumento en 159 veces (IC del 95 %: 27,2–938,2) del riesgo entre las personas con una variante inactiva de la enzima aldehído deshidrogenasa. La aplicación de estas estimaciones de la respuesta en relación con la dosis al perfil nacional del consumo de alcohol dio lugar a la notificación de 5279 muertes de cáncer esofágico y 102 988 AVAD perdidos, lo equivale a casi el doble de las estimaciones producidas por la carga mundial más reciente de la actividad de la enfermedad.

**Conclusión:**

El uso de datos mundiales sobre la respuesta en relación con la dosis da lugar a una subestimación de la carga de enfermedad de cáncer esofágico en Japón. Siempre que sea posible, los estudios sobre la carga nacional de la enfermedad deben utilizar los resultados de la población afectada.

## ملخص

**الغرض:**

تنقيح تقديرات عبء سرطان المريء ذي الصلة بتعاطي الكحول في اليابان.

**الطريقة:**

أجرينا بحثاً في قاعدة بيانات PubMed عن الاستعراضات المنشورة والدراسات الأصلية حول تعاطي الكحول وتعدد أشكال نازعة هيدروجين الألدهيد واختطار سرطان المريء في اليابان، التي تم نشرها قبل عام 2014. وأجرينا تحليلات وصفية للتأثيرات العشوائية، بما في ذلك تحليلات الفئات الفرعية حسب متغيرات نازعة هيدروجين الألدهيد. وقمنا بتقدير حالات الوفاة وفقدان سنوات العمر المصححة باحتساب مدد العجز جراء الإصابة بسرطان المريء باستخدام توزيعات التعرض للكحول استناداً إلى السن والجنس والمخاطر النسبية لكل وحدة تعرض.

**النتائج:**

قمنا بتحديد 14 دراسة ذات صلة. واشتملت ثلاث دراسات أترابية وأربع دراسات لحالات إفرادية مقترنة بحالات ضابطة على بيانات عن علاقة الجرعة بالاستجابة. وأظهرت البيّنات المستمدة من الدراسات الأترابية تعرض الأشخاص الذين تعاطوا ما يعادل 100 غم/يوم من الكحول الصافي إلى خطورة الإصابة بسرطان المريء بمقدار 11.71 مرة (فاصل الثقة 95 %؛ فاصل الثقة: من 2.67 إلى 51.32) مقارنة بالذين لم يتعاطوا الكحول إطلاقاً. وأظهرت البيّنات المستمدة من دراسات الحالات الإفرادية المقترنة بحالات ضابطة أن الزيادة في الخطورة بلغت 33.11 مرة (فاصل الثقة 95 %؛ فاصل الثقة: من 8.15 إلى 134.43) في عامة السكان. ويتضح الفرق حسب تصميم الدراسة في الخطورة التي بلغت 159 مرة (فاصل الثقة 95 %؛ فاصل الثقة: من 27.2 إلى 938.2) بين الأشخاص الذين لديهم متغير غير فعال لإنزيم نازعة هيدروجين الألدهيد. ونتج عن تطبيق هذه التقديرات لعلاقة الجرعة بالاستجابة على المرتسم الوطني لتعاطي الكحول 5279 حالة وفاة بسرطان المريء وفقدان 102988 من سنوات العمر المصححة باحتساب مدد العجز - وهو تقريباً ضعف التقديرات الناتجة عن العبء العالمي الأحدث لممارسة المرض.

**الاستنتاج:**

ينتج عن استخدام بيانات علاقة الجرعة بالاستجابة على الصعيد العالمي التقليل من تقدير عبء المرض الناجم عن سرطان المريء في اليابان. وينبغي أن تستخدم دراسات عبء المرض على الصعيد الوطني النتائج المستمدة من السكان المعنيين حسبما أمكن.

## 摘要

**目的:**

得到更精确的日本酒精性食道癌负担估算值。

**方法:**

我们搜索PubMed数据库，查找2014年前发表的关于日本酒精摄入、乙醛脱氢酶多态性以及食道癌风险的综述与原始研究。我们还进行了随机影响综合分析，包括用乙醛脱氧酶变体进行的亚组分析。根据酒精影响类别的年龄、性别及相对风险，我们使用酒精影响分布估算了食道癌死亡率与残疾调整生命年（DALY）的损失。

**结果:**

我们找到了14项相关研究。三项队列研究和四项病例对照研究提供剂量反应的数据。队列研究的证据表明，与从不饮酒的人群相比较，这些每天消耗相当于100克纯酒精的人群患食道癌的风险是前者的11.71倍（95%置信区间，CI：2.67-51.32）。病例对照研究的证据表明，从整体人口方面看，患食道癌的风险增加了33.11倍（95%置信区间，CI：8.15-134.43）。研究设计发现的差异显示，携带不活跃乙醛脱氢酶变体的人患食道癌的风险是159倍（95%，CI：27.2-938.2）。把这些剂量反应数值应用到全国酒精摄入数据中，我们得出食道癌死亡人数是5279人，残疾调整生命年损失是102988年，这些数据几乎是最新全球疾病负担研究数据的两倍。

**结论:**

使用全球剂量反应数据导致对日本食道癌疾病负担的低估。在可能的情况下，全国疾病负担研究应该使用相关人群的研究结果。

## Резюме

**Цель:**

Выполнить точную оценку бремени рака пищевода, обусловленного употреблением алкоголя, в Японии.

**Методы:**

Мы изучили содержащуюся в базе данных PubMed информацию по опубликованным до 2014 г. обзорам и оригинальным исследованиям, касающимся употребления алкоголя, полиморфизмов альдегиддегидрогеназы и риска развития рака пищевода в Японии. Мы провели метаанализ случайных эффектов, включая анализы в подгруппах по вариантам альдегиддегидрогеназы. Мы оценили количество смертей и утраченных лет жизни с поправкой на инвалидность (ГЖПИ) в результате рака пищевода, используя распределение подвергающихся воздействию алкоголя лиц по возрасту, полу и относительному риску на единицу воздействия.

**Результаты:**

Мы выявили 14 исследований, имеющих отношение к вопросу. Для трех исследований в когортах и четырех исследований методом «случай-контроль» были представлены данные о зависимости между дозой и эффектом. Данные когортных исследований свидетельствуют о том, что у лиц, потреблявших алкоголь в дозе, эквивалентной 100 г чистого алкоголя в сутки, риск развития рака пищевода возрастает в 11,71 раз (доверительный интервал, ДИ 95%: 2,67–51,32) по сравнению с лицами, никогда не употреблявшими алкоголь. Данные исследований методом «случай-контроль» свидетельствуют о том, что кратность повышенного риска развития заболевания во всей популяции составляет 33,11 (ДИ 95%: 8,15–134,43). Разница в плане исследования объясняется кратностью риска 159 (ДИ 95%: 27,2–938,2) для лиц с неактивным вариантом фермента альдегиддегидрогеназы. Применение таких оценок данных о зависимости между дозой и эффектом к национальному профилю потребления алкоголя позволило получить 5 279 смертей от рака пищевода и 102 988 утраченных ГЖПИ, что почти в два раза превышает результаты последних международных оценок бремени данной болезни.

**Вывод:**

Использование данных о зависимости между дозой и эффектом, полученных по результатам международных оценок, приводит к недооценке бремени рака пищевода в Японии. В ситуациях, когда это возможно, для исследований по расчету бремени заболевания на уровне страны следует использовать результаты, касающиеся конкретной популяции.

## Introduction

Alcohol consumption is a major contributor to the global burden of disease^1^^,^^2^ and is a major risk factor for cancer.^3^^–^^6^ Of all alcohol-related cancers, oesophageal has the highest alcohol-attributable fraction^6^ – i.e. the highest proportion of these cancers would be prevented if no alcohol were consumed.^6^^–^^8^ The global burden of disease (GBD) study estimates that in 2010 alcohol-attributable oesophageal cancer resulted in 76 700 deaths and 1 825 000 disability adjusted life years (DALYs) lost, globally.^9^

A large portion of oesophageal cancers attributable to alcohol consumption occur in Asian countries – 52.2% (40 000) of all alcohol-attributable oesophageal cancer deaths and 51.8% (945 000) of all alcohol-attributable oesophageal cancer DALYs. The alcohol-attributable portions for countries in this region have been calculated based on global meta-analyses.^10^^,^^11^ However, this assumes that the alcohol-attributable risk for oesophageal cancer is the same in all regions. Preliminary evidence, on the other hand, shows that the risk for this cancer is different for people of Asian origin, because of genetic polymorphisms – most importantly the aldehyde dehydrogenase 2 (*ALDH2*) and alcohol dehydrogenase 1B (*ADH1B*) polymorphisms.^12^^–^^15^ Thus, the real risk and burden of alcohol-attributable oesophageal cancer in Asia may have been underestimated.

In Japan in 2010, oesophageal cancer was among the top 20 causes of years of life lost (11 deaths and 181 DALYs per 100 000 people per year).^9^ We did a systematic review and meta-analyses of studies conducted in the Japanese population to estimate the alcohol-attributable burden of oesophageal cancer. We then compared these estimates to the GBD 2010 estimates.^1^ We also estimated risk functions according to *ALDH2* subsets and investigated potential interactions between *ALDH2* and *ADH1B* polymorphisms.

## Methods

### Data search and selection

We followed the Preferred Reporting Items for Systematic Reviews and Meta-analyses guidelines.^16^ We used the latest editions of the International Agency for Research on Cancer (IARC) monographs on alcohol^3^^,^^4^ to identify potentially eligible studies. Additionally, we searched PubMed for publications published before 2014. We did two searches using the following search terms; Search 1: “cancer or neoplasm or carcinom*” and “*ALDH2* or *ADH1B* or *ADH2* or *ADH3* or *ADH1C* or dehydrogenase*” and “alcohol or ethanol”; Search 2: “alcohol or ethanol” and “cohort” and “cancer” and “japan” and “review” and “mortality”. Inclusion criteria for analyses investigating the relationship between alcohol consumption, *ALDH2*, and oesophageal cancer were: (i) prospective or historical cohort or case-control study design; (ii) a measure of risk and its corresponding measure of variability was reported or there were sufficient data for us to calculate these; (iii) oesophageal cancer was reported as a separate outcome; (iv) data on total alcohol intake for at least two exposure categories among current drinkers, or estimates for *ALDH2* variants by alcohol intake were reported; (v) risk estimates were at least age-adjusted; and (vi) the study was conducted in Japan after 1980. In addition, we searched reference lists of identified articles for additional articles. No active filters or language restrictions were applied. We excluded measures of pure drinking frequency and qualitative characteristics – such as social or problem drinker. Oesophageal cancer cases (*International Classification of Diseases* [ICD] version 9: 150, ICD-10: C15) were defined as newly diagnosed at the first visit to a specialized clinic, through cancer registries or cause of death on death certificates.

Most quality scores for primary studies are tailored for meta-analyses of randomized trials of interventions^17^^–^^19^ and many criteria for such scores do not apply to epidemiological studies examined in this study. Additionally, quality score use in meta-analyses remains controversial.^19^^,^^20^ As a result, we included quality components in the inclusion and exclusion criteria of the systematic search and separate meta-analyses – such as study design and alcohol measurement – and conducted subgroup analyses based on study design and genetic polymorphisms.

### Data extraction

From all relevant articles we extracted: authors’ names, year of publication, country, calendar year(s) of baseline examination, follow-up period, setting, assessment of oesophageal cancer diagnosis, range of age at baseline, sex, number of observed oesophageal cancer cases among participants by alcohol exposure category, number of total participants by alcohol exposure category, adjustment for potential confounders and effect size with its standard error. We used the most fully adjusted effect size reported and selected estimates where lifetime abstainers were used as the risk reference group when those were available. Assessment of full-text articles with uncertain eligibility and data abstraction were conducted independently by two authors who discussed differences until consensus was reached. When there was not enough information presented in the article, we contacted the corresponding author.

We converted alcohol intake into grams of pure alcohol per day (g/day) using the midpoints (mean) of reported categories in the studies. For open-ended categories of alcohol intake, we added three-fourths of the previous category’s range to the lower bound of the open-ended categories. We used reported conversion factors in the studies when standard drinks were the unit of measurement. Hazard ratios and odds ratios were assumed to be equivalent to relative risks (RR). We used fractional polynomials^21^ to derive the best fitting function for average alcohol consumption in g/day using the pool-first approach described by Orsini et al.^22^ Linear and first-degree models were estimated using the following range of powers: −2, −1, 0, 1, 2, 3.^21^ Significant increases in deviance were determined by likelihood ratio tests with one degree of freedom.

### Data analyses

We conducted several meta-analyses and used the most comprehensive data available separately for each analysis when multiple reports from the same cohort were published. For studies providing data on two or more alcohol intake categories among current drinkers, we pooled data from (i) cohort studies; (ii) case-control studies; (iii) case-control studies that provided stratified data by *ALDH2* variants. We conducted sensitivity analyses on the interaction between variants of *ADH1B* within the genetic variants of *ALDH2.* In analyses investigating *ALDH2* variants, studies were pooled separately for the active variant (ALDH2*1/*1) and inactive variants (ALDH2*1/*2 and ALDH2*2/*2). No cohort studies provided *ALDH2* genotype data. Where possible, we avoided ALDH2*2/*2 variants because of the low number of cases. No systematic information on the distribution of *ALDH2* variants by drinking level was available and we therefore used the distribution of drinking by *ALDH2* variants among controls in case-control studies to estimate this distribution at the population level. Finally, studies were pooled using DerSimonian-Laird random-effect models to allow for between-study heterogeneity.^23^ Variation in the effect size other than chance because of heterogeneity between studies was quantified using the *I^2^* statistic.^24^ We conducted meta-regression analyses to identify study characteristics that influenced the association between alcohol consumption and oesophageal cancer risk. Because of few available studies, we were only able to investigate study design in such meta-regression analyses. Examination of potential publication bias using Egger’s regression-based test^25^ was planned, but was not done because of the few studies included. All meta-analyses were conducted on the natural log scale in Stata statistical software, version 12.1 (StataCorp. LP, College Station, United States of America) and *P* < 0.05 (two-sided) was considered statistically significant.

We estimated deaths and DALYs lost from oesophageal cancer attributable to alcohol consumption in Japan applying a standardized alcohol-attributable fraction method^26^ using the statistical software package R, version 3.0.3 (R Foundation for Statistical Computing, Vienna, Austria). These deaths and DALYs were estimated by comparing the risk difference of oesophageal cancer under current conditions compared to the risk of oesophageal cancer under the theoretical-minimum-risk exposure scenario where no one has consumed alcohol.^1^^,^^7^ These calculations combine information on the prevalence of alcohol consumption adjusted for per capita consumption and RRs for oesophageal cancer. Lifetime abstainers were used as the reference group and compared to former drinkers and current drinkers – by average daily alcohol consumption. Data on alcohol drinking status were obtained from the 2010 GBD study,^1^ where data on drinking status were based on data from large population surveys. Data on per capita consumption were from the Global Information System on Alcohol and Health.^27^ Calculations for Japan were based on RRs from this study and RRs for GBD estimates for Japan were based on Corrao et al.^11^

## Results

**Fig. 1:**
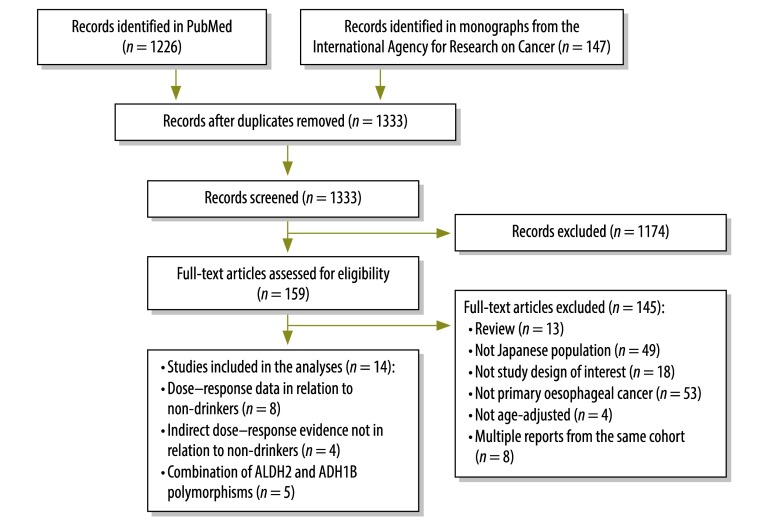
Flowchart for the selection of studies on alcohol consumption and oesophageal cancer in Japan

ADH1B: alcohol dehydrogenase 1B; ALDH2: aldehyde dehydrogenase 2.

After removal of duplicates, we evaluated 1333 records for inclusion in our study. Based on titles and abstracts, we excluded 1174 articles and screened 159 in full-text articles (Fig. 1). After excluding duplicate reports of the same cohorts, we analysed 11 case-control studies^28^^–^^38^ and 3 cohort studies.^39^^–^^41^ Eight case-control and cohort studies^32^^–^^36^^,^^39^^–^^41^ reported estimates for at least two alcohol intake categories in comparison to non-drinkers. These studies were used for a nonlinear dose–response analysis of oesophageal cancer risk, including stratified analyses by *ALDH2* variants. Four case-control studies^28^^–^^31^ provided indirect evidence for only one alcohol intake category Table 1 (available from: http://www.who.int/bulletin/volumes/93/5/14-142141). In total five studies had data on *ALDH2* and *ADH1B* variants stratified or adjusted by level of alcohol consumption.^29^^,^^31^^,^^36^^–^^38^

**Table 1:** Summary of studies assessing the relationship between alcohol and oesophageal cancer in Japan

Study and year	Study design (follow-up)	Setting	Study period, age and sex	No. of cases and controls	Case and control identification	Alcohol assessment	Adjustment
Yokoyama et al., 1998^28^	Case-control	National Institute on Alcoholism, Kurihama National Hospital	1987–1997, ≥ 40 years, men	Cases: 87, whereof ALDH2*1/*1: 41, ALDH2*1/*2 and ALDH2*2/*2: 46 Controls: 487	Cases: SCC histologically diagnosed at alcohol treatment entry or before onset of alcoholism Controls: cancer-free alcoholics	Alcohol dependence (DSM-III), mean alcohol intake 123 g/day	Age at admission to alcohol treatment, daily alcohol consumption, number of cigarettes
Takezaki et al., 2000^35^	Case-control	Aichi Cancer Centre	1988–1997, 40–79 years, men	Cases: 284 Controls: 11 384 (former alcohol drinkers were excluded in the analysis)	Cases: first out-visit outpatients diagnosed with primary cancer of the oesophagus (ICD-9: 150, ICD-10: C15) Controls: first-visit outpatients confirmed to be cancer-free (including no history of cancer assessed by questionnaire)	Drinking levels: never or occasionally, former drinkers, current drinkers < 1.5 drinks/day, ≥ 1.5 drinks/day. One drink = 1 *go* (Japanese sake with 27 mL ethanol)	Age, year and season of visit, smoking (never, former, current, < 30 and ≥ 30 pack-years), consumption of raw vegetables
Yokoyama et al., 2001^29^^,a^	Case-control	National Institute on Alcoholism, Kurihama National Hospital	1993–2000, ≥ 40 years, men	Cases: 112, whereof ALDH2*1/*1: 50, ALDH2*1/*2 and ALDH2*2/*2: 62 Controls: 526	Cases: SCC histologically diagnosed at alcohol treatment entry or before onset of alcoholism Controls: cancer-free alcoholics	Alcohol dependence (DSM-III), mean alcohol intake 123 g/day	Age at admission to alcohol treatment, daily alcohol consumption, number of cigarettes
Matsuo et al., 2001^30^	Case-control	Aichi Cancer Centre	1984–2000, 40–76 years, women and men	Cases: 102, whereof ALDH2*1/*1: 35, ALDH2*1/*2: 66, ALDH2*2/*2: 1 Controls: 241	Cases: first diagnosis for oesophageal cancer Controls: first-visit outpatients confirmed by gastroscopy to have no oesophagus or stomach cancer	Drinking status (2 categories): > 3 *go* (Japanese sake with 75 mL pure alcohol)/day ≥ 5 times per week, and all others (non-drinkers and drinkers with ≤ 3 *go* per day and < 5 times per week)	Age, smoking (never, former, current, < 30 and ≥ 30 pack-years), consumption of raw vegetables
Yokoyama et al., 2002^36^^,a^	Case-control	National Cancer Centre Hospital, National cancer Centre Hospital East, Kawasaki Municipal Hospital, National Osaka Hospital	2000–2001, 40–79 years, men	Cases: 220 SCC, whereof ALDH2*1/*1: 60, ALDH2*1*2: 160 Controls: 590	Cases: SCC newly diagnosed by histology within 3 years before registration in study Controls: cancer-free men who visited two Tokyo clinics for annual health check-up	Drinking levels: non- or rare drinkers, former drinkers, current drinkers 1–8.9 U/week, 9–17.9 U/week, ≥ 18 U/week. U = unit of alcohol (1 serving of sake, 22 g pure alcohol/U)	Age, frequency of drinking strong alcoholic beverages, smoking (pack years), intake frequency of green-yellow vegetables, intake frequency of fruits
Yokoyama et al., 2003^32^	Case-control	National Cancer Centre Hospital, National cancer Centre Hospital East, Kawasaki Municipal Hospital, National Osaka Hospital	2000–2001, 40–79 years, men	Cases: 220 SCC Controls: 598 (former alcohol drinkers were excluded in the analysis)	Cases: SCC newly diagnosed by histology within 3 years before registration in study Controls: cancer-free men who visited two Tokyo clinics for annual health check-ups	Drinking levels: non- or rare drinkers, former drinkers, current drinkers 1–8.9 U/week, 9–17.9 U/week, ≥ 18 U/week. U = unit of alcohol (1 serving of sake, 22 g pure alcohol/U)	Age, frequency of drinking strong alcoholic beverages, smoking (pack years), intake frequency of green-yellow vegetables, intake frequency of fruits
Nakaya et al., 2005^40^	Cohort (7 years follow-up)	Miyagi II	1990–1997, 40–64 years, men	Cases: 48 among 19 607 participants (former alcohol drinkers were excluded in the analysis)	Cases were identified via record linkage to cancer registry	Drinking levels: five categories based on drinking frequency and amount per occasion: never, former-drinkers, current drinkers < 22.8 g pure alcohol/day, 22.8–45.5 g/day, and ≥ 45.6 g/day	Age, smoking (never, former, current 1–19 cigarettes per day, 20–29 per day, 30 or more per day), education, daily consumption of orange and other fruit juice, spinach, carrot or pumpkin, and tomato
Yokoyama et al., 2006^33^	Case-control	National Cancer Centre Hospital, National cancer centre Hospital East, Kawasaki Municipal Hospital, National Osaka Hospital	2000–2004, 40–79 years, women	Cases: 43 SCC, whereof ALDH2*1/*1: 25, ALDH2*1/*2: 18 Controls: 365	Cases: SCC newly diagnosed by histology within 3 years before registration in study Controls: cancer-free women who visited two Tokyo clinics for annual health check-up	Drinking levels: non- or rare drinkers, former drinkers, current drinkers 1–8.9 U/week, 9–17.9 U/week, ≥ 18 U/week. U = unit of alcohol (1 serving of sake, 22 g pure alcohol/U)	Age, smoking (pack years), intake frequency of green-yellow vegetables, intake frequency of fruits, preference for hot food or drinks
Ozasa et al., 2007^41^	Cohort (not reported)	JACC	1988–1990, 40–79 years, men	Cases: 117 among 42 578 participants (former alcohol drinkers were excluded)	Death certificates (ICD-10: C15)	Drinking levels: non- or rare drinkers, former drinkers, current drinkers < 54 mL pure alcohol/day, 54–80 mL/day, ≥ 81 mL/day	Age, study area
Cui et al., 2009^31^^,a^	Case-control	Biobank Japan	2003–2008, 35–85 years, men and women	Cases: 1 066, whereof ALDH2*1/*2: 735, ALDH2*1/*1 and ALDH2*2/*2: 331 Controls: 2 761	Cases: histologically diagnosed SCC Controls: volunteers or registered in Biobank for diseases other than cancer	Drinking status: none/rare (0–96.5 g pure alcohol/week), and other drinkers (≥ 96.5 g/week)	Age, gender (analyses among heavy alcohol consumers, > 96.5 g/week)
Ishiguro et al., 2009^39^	Cohort (14 years follow-up)	JPHC I+II	1990 and 1993, 40–59 years, men	Cases: 215 SCC among 60 876 participants	Active patient notification from hospital and linkage to Cancer Registry (ICD-0–3: C15.0–15.9)	Drinking levels: non-drinkers, less than weekly drinking (frequency only), 1–149 g pure alcohol /week, 150–299 g/week, ≥ 300 g/week	Age, study area, body mass index, preference for spicy food and drinks, smoking status (never, past, current), flushing response
Oze et al., 2010^34^	Case-control	Aichi Cancer Centre	2001–2005, ≥ 18 years, men and women	Cases: 260, whereof ALDH2*1/*1: 67, ALDH2*1/*2 and ALDH2*2/*2: 198 Controls: 487	Cases: first out-visit outpatients diagnosed with primary cancer of the oesophagus (ICD-10: C15) Controls: First-visit outpatients confirmed to be cancer-free (including no history of cancer assessed by questionnaire)	Drinking levels: never, moderate (≤ 4 days/week), high-moderate (≥ 5 days/week and < 46 g pure alcohol/occasion), heavy (≥ 5 days/week and ≥ 46 g/occasion)	Frequency matched by age group (< 40, 40–49, 50–59, 60–69, ≥ 70 years) and sex. Adjustment for cumulative smoking, facial flushing, fruit and vegetable intake, frequent intake of hot beverages and body mass index
Yang et al., 2005^37^^,a^	Case-control	Aichi Cancer Centre	2000–2004, 18–79 years, men and women	Cases: 165, whereof ALDH2*1/*1: 38, ALDH2*1/*2 and ALDH2*2/*2: 127 Controls: 495	Cases: histologically diagnosed with primary cancer of the oesophagus (159 SCC, 6 adenocarcinomas) Controls: first-visit outpatients confirmed to be cancer-free (including no history of cancer assessed by questionnaire)	Drinking levels: non-drinker, non-heavy drinkers (< 5 drinking days/week and < 50 g pure alcohol/occasion) and heavy drinkers (drinking ≥ 5 days/week and ≥ 50 g pure alcohol/occasion) were adjusted for in regression model as reported	Age, sex, smoking, drinking
Tanaka et al., 2010^38^^a^	Case-control	Juntendo University Hospital, National Cancer Center Hospital, Kurume University Hospital, Saitama Cancer Center, Kagoshima University Hospital, Kyushu University Hospital	2000–2008, 35–85 years, men and women	Cases: 742, whereof ALDH2*1/*1: 194, ALDH2*1/*2 and ALDH2*2/*2: 548 Controls: 820	Cases: pathologically newly diagnosed SCC Controls: healthy controls without cancer history recruited from Kyushu University and Hospital and related hospitals	Drinking levels: Non-drinker and ever drinkers	Sex, age, study area

ADH1B: alcohol dehydrogenase 1B; ALDH2: aldehyde dehydrogenase 2; DSM: diagnostic statistical manual; ICD: International Classification of Diseases; JACC: Japan collaborative cohort study; JPHC: Japan public health centre-based prospective study; SCC: squamous cell carcinoma of the oesophagus.

^a^ Included in the sensitivity analysis on interaction between *ALDH2* and *ADH1B* on oesophageal cancer risk in Japan.

Note: ALDH2*2/*2 was excluded in our analyses where possible because of the low number of cases.

**Fig. 2:**
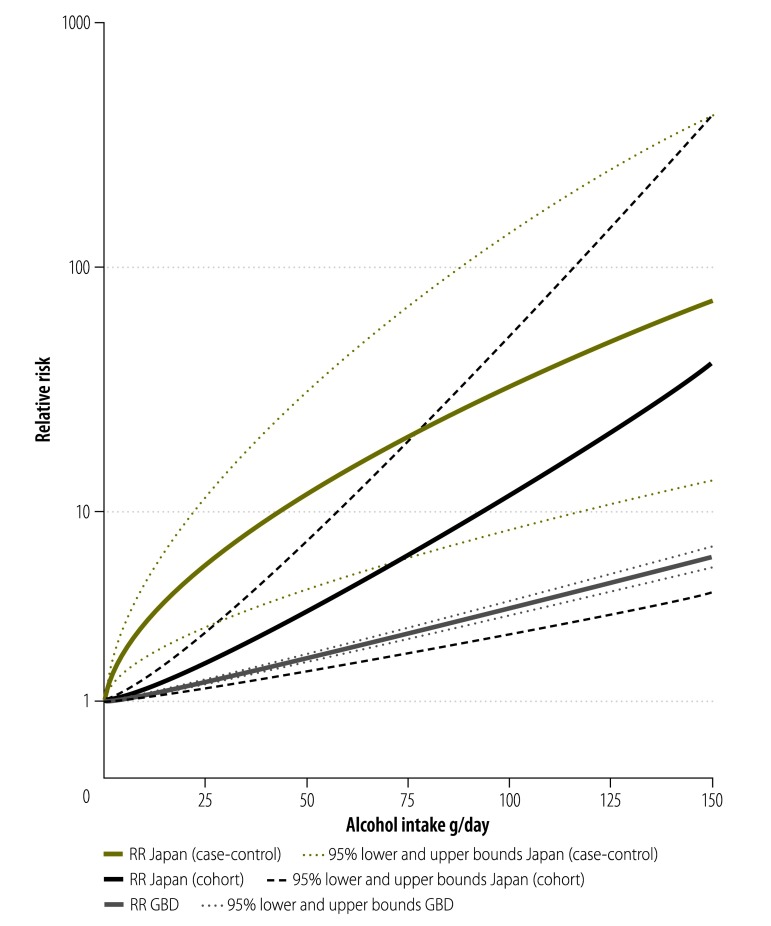
Risk curves for alcohol consumption and oesophageal cancer risk based on Japanese studies or the Global Burden of Disease 2010 study

GBD: Global Burden of Disease; RR: relative risk.

As shown in Fig. 2, the risk for oesophageal cancer identified in cohort studies from Japan^39^^–^^41^ was higher compared with the most recent GBD estimate (RR: 11.71; 95% confidence interval, CI: 2.67–51.32 and RR: 3.59; 95% CI: 3.34–3.87, respectively at 100 g/day of pure alcohol intake). The risk identified in case-control studies^32^^–^^35^ (RR: 11.88; 95% CI: 4.41–31.99 at 50 g/day of pure alcohol intake; RR: 33.11; 95% CI: 8.15–134.43 at 100 g/day of pure alcohol intake) was much higher than the Japanese cohort studies or GBD estimates. In a meta-regression, the difference between case-control studies and cohort studies was significant (*P* = 0.014). We observed moderate heterogeneity among cohort studies (*I^2^* = 60%, *P* = 0.082), and high heterogeneity among case-control studies (*I^2^* = 89%, *P* < 0.001).

**Fig. 3:**
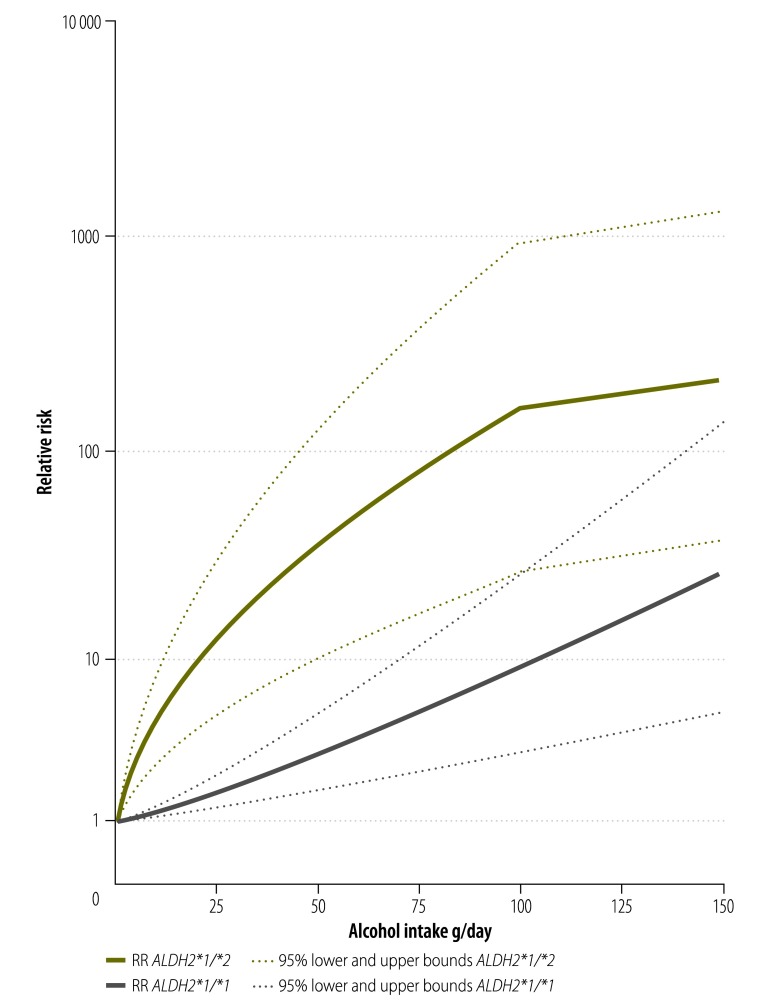
Risk curves for alcohol consumption and oesophageal cancer risk based on aldehyde dehydrogenase 2 polymorphisms, Japan

ALDH2: aldehyde dehydrogenase 2; RR: relative risk.

Note: ALDH2*1/*1 corresponds to an active enzyme and ALDH2*1/*2 corresponds to an inactive enzyme.

The risk curves by *ALDH2* variants in Japan are displayed in Fig. 3. Three case-control studies^33^^,^^34^^,^^36^ provided dose–response data for an investigation of *ALDH2* polymorphisms in reference to non-drinkers: ALDH2*1/*2 (372 cases) and ALDH2*1/*1 (151 cases). Inactive variants of ALDH*2* enzyme showed markedly higher risks with increasing alcohol consumption. The RR compared to non-drinkers was 36.15 (95% CI: 10.34–126.40) at 50 g/day of pure alcohol and 159 (95% CI: 27.2–938.2) at 100 g/day of pure alcohol intake among people carrying the ALDH2*1/*2 variant. In comparison, the RR among those carrying the ALDH2*1/*1 variant was 2.99 (95% CI: 1.75–5.12) at 50 g/day of pure alcohol intake and 8.94 (95% CI: 3.05–26.23) at 100 g/day of pure alcohol. Based on two studies that included people with alcohol dependence (median 120 g/day of pure alcohol intake), people with the inactive variant of *ALDH2* had an RR of 13.00 (95% CI: 8.99–18.80) compared to those with the active variant.^28^^,^^29^ We interpolated this difference in risk in the curve for ALDH2*1/*2 in Fig. 3, and held the risk increase among people with this *ALDH2* variant constant beyond 100 g/day of pure alcohol intake because there were insufficient data to reliably estimate this risk function. Once case-control studies were stratified by *ALDH2* variant, there was little or no heterogeneity (ALDH2*1/*1, *I^2^* = 0%, *P* = 0.78; ALDH2*1/*2, *I^2^* = 44%, *P* = 0.17). Another two studies,^30^^,^^31^ although they did not provide data in reference to non-drinkers, were in close agreement with our reported risk functions.

**Fig. 4:**
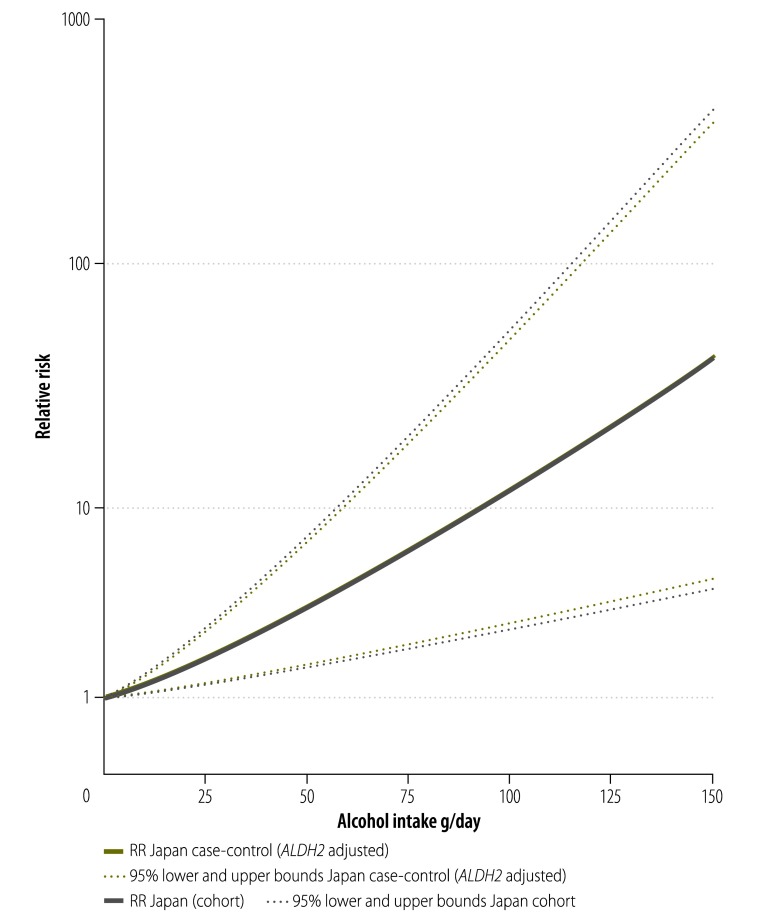
Risk curves for alcohol consumption and oesophageal cancer risk adjusted for aldehyde dehydrogenase 2 polymorphisms, Japan

ALDH2: aldehyde dehydrogenase 2; RR: relative risk.

Notes: ALDH2*1/*1 corresponds to an active enzyme and ALDH2*1/*2 corresponds to an inactive enzyme. The relative risk curves by study design overlap almost completely after adjustment for drinking levels at the population level by ALDH2 variants.

**Fig. 5:**
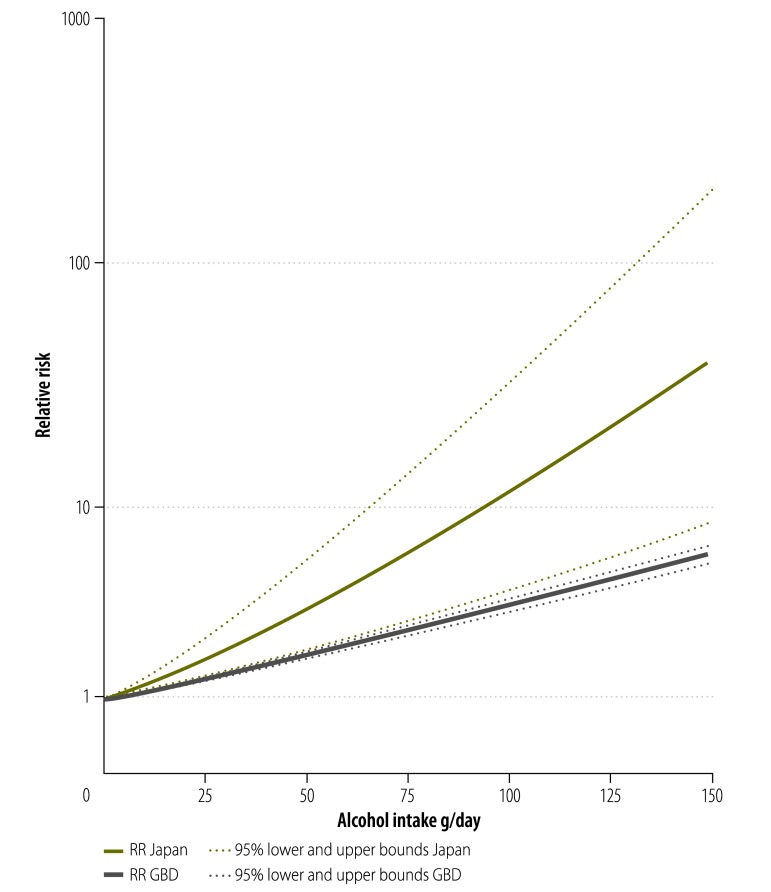
Risk curves for alcohol consumption and oesophageal cancer risk based on Japanese studies adjusted for aldehyde dehydrogenase 2 polymorphisms and the Global Burden of Disease 2010 study

ALDH2: aldehyde dehydrogenase 2; RR: relative risk.

With regard to differences in risk curves by study design, Table 2 shows that among case-control studies with multiple alcohol intake categories, 72% (350/483) of oesophageal cancer cases among drinkers occurred in 32% (313/980) of the drinking population, namely individuals with the genetic variant ALDH2*1/*2. When the risk curves from case-control studies (Fig. 3) were combined (weighted by their distribution of alcohol consumption by *ALDH2* variants at the population level) the risk functions from case-control and cohort studies almost entirely overlapped (Fig. 4). Combining adjusted case-control and cohort studies, at 100 g/day pure alcohol intake, the risk in Japan was markedly elevated (RR: 11.65, 95% CI: 4.16–32.62) compared to GBD estimates (RR: 3.55, 95% CI: 3.30–3.82) (Fig. 5).

**Table 2:** Distribution of alcohol consumption and the *ALDH2* polymorphism in individuals with oesophageal cancer and study controls, Japan

Polymorphism	Alcohol consumption, No*.* of individuals (%)				
	Non-drinker	0–25 g/day	> 25–75 g/day	> 75 g/day	Total
**Controls**					
ALDH2*1/*2	336 (52)	234 (36)	51 (8)	28 (4)	649 (100)
ALDH2*1/*1	145 (18)	350 (43)	231 (28)	86 (11)	812 (100)
**Oesophageal cancer cases**					
ALDH2*1/*2	22 (6)	94 (25)	180 (48)	76 (20)	372 (100)
ALDH2*1/*1	18 (12)	35 (23)	61 (40)	37 (25)	151 (100)

ALDH2: aldehyde dehydrogenase 2.

Note: The ALDH2*1/*1 corresponds to an active enzyme and ALDH2*1/*2 corresponds to an inactive enzyme.

Data sources: Yokoyama et al.^36^ Yokoyama et al.^33^ Oze et al.^34^

**Fig. 6:**
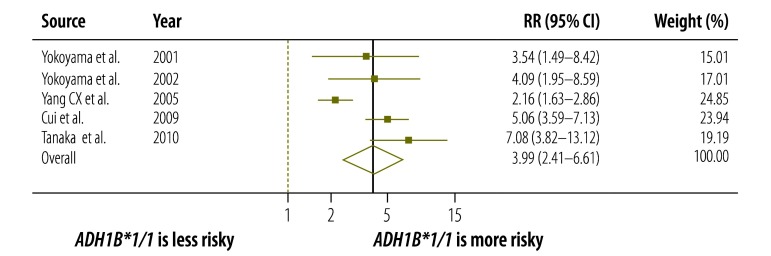
Relationship between alcohol dehydrogenase 1B polymorphisms and oesophageal cancer risk in Japanese with inactive aldehyde dehydrogenase 2

ADH1B: alcohol dehydrogenase 1B; CI: confidence interval; RR: relative risk.

**Fig. 7:**
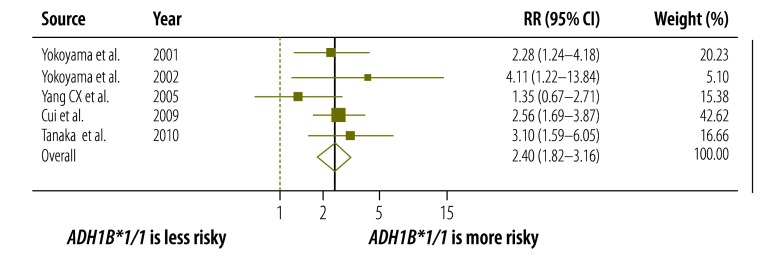
Relationship between alcohol dehydrogenase 1B polymorphisms and oesophageal cancer risk in Japanese people with active aldehyde dehydrogenase 2

ADH1B: alcohol dehydrogenase 1B; CI: confidence interval; RR: relative risk.

Note: ADH1B*1/*1 corresponds to the slow-acting form of the enzyme and ADH1B*1/*2 and ADH1B*2/*2 corresponds to the fast-acting form.

To investigate the interaction between *ALDH2* and *ADH1B* gene variants, we performed a sensitivity analysis using five of the 11 identified case-control studies.^29^^,^^31^^,^^36^^–^^38^ Regardless of *ALDH2* variant, the pooled RRs were higher for Japanese with the slow-acting ADH1B*1/*1 variant than for Japanese with the fast-acting ADH1B*1/*2 or *2/*2 variants. The RR was 3.99 (95% CI: 2.41–6.61; I^2^ = 81%, *P* < 0.001) and 2.40 (95% CI: 1.92–3.16; I^2^ = 1%, *P* = 0.40) for individuals with an inactive and active ALDH2, respectively (Fig. 6 and Fig. 7). 

Using our calculated risk relations for alcohol-attributable oesophageal cancer results in almost twofold higher estimates for deaths (5279) and DALYs lost (102 988) compared with the current GBD estimates (2749 and 53 826, respectively; Table 3). These results are irrespective of whether the estimates were based on cohort studies or on case-control studies, in each case adjusted for population prevalence of genotypes.

**Table 3:** Estimated mortality and burden of disease for alcohol-attributable oesophageal cancer in Japan 2010

Estimate	Women			Men			Total	
	No. of deaths	No. of DALYs		No. of deaths	No. of DALYs		No. of deaths	No. of DALYs
GBD 2010	202	3 089		2 547	50 737		2 749	53 826
Japanese cohort studies	346	5 498		4 925	97 284		5 271	102 782
Japanese adjusted case-control studies	346	5 514		4 933	97 474		5 279	102 988

DALYs: disability-adjusted life-years; GBD: Global Burden of Disease.

## Discussion

For Japan, we estimated a twofold higher mortality and burden of disease using risk functions derived from Japanese populations compared with the 2010 GBD estimates, which are based on global risk functions. We obtained separate estimates based on independent methods – either Japanese cohort data or adjusted Japanese case-control studies – and these estimates were comparable. This strengthens our conclusion that the current GBD method underestimates Japanese oesophageal cancer outcomes. Since we took into consideration genetic polymorphisms commonly observed in the Japanese population, we would predict a similar degree of underestimation for alcohol-attributable oral, pharynx and larynx cancers in Japan.^34^^,^^42^^,^^43^ Furthermore, the burden of other alcohol-attributable cancers where acetaldehyde plays an important role might be underestimated.^3^^,^^4^

We found that the slow-acting *ADH1B* variant also increased the risk for oesophageal cancer, regardless of *ALDH2* variant. However, the slow-acting variant is only present in 6% of the Japanese population,^44^ whereas 90% of Caucasians carry the variant.^45^ As we had restricted all our analyses to Japanese individuals, the potential protective effect of the fast-acting ADH1B*1/*2 or *2/*2 variants has been already included. Similarly, risk estimates from cohort studies should not be affected by the differential risk curves for *ALDH2* and *ADH1B* variants and their combinations if the prevalence of each combination of polymorphisms is reflected in the sample.

The study has some limitations. First, any systematic review or meta-analysis is only as good as the literature it is based on. Although case-control studies initially showed high heterogeneity as measured by *I^2^* values indicating potential bias, there was little heterogeneity after these studies were stratified by *ALDH2* variants. Second, while the procedures to estimate alcohol-attributable fractions are standard,^26^^,^^27^^,^^46^ subsequent adjustments to survey results may bias consumption in either direction.^47^^–^^49^ However, as the same method of triangulation of surveys and per capita estimation was applied to GBD and to national estimates,^1^ the comparison between these estimates should be valid. Finally, the estimates of attributable risk and burden of disease for heavy alcohol intake are based on few studies and thus may be biased.^50^ By including risk estimates of people with alcohol dependence, we attempted to minimize this bias.

While there may be some biases in our quantitative estimates of alcohol-attributable burden for oesophageal cancer, they still show that global estimates underestimate the burden in Japan. This will likely be true for GBD estimates for China and the Republic of Korea as well, where a considerable proportion of the population also carry the inactive *ALDH2* allele, (34% and 29%, respectively).^44^ In populations with a high proportion of these polymorphisms, studies based on global dose response data are likely to underestimate many alcohol-attributable cancers.

Efforts should be made to estimate country-specific risks for diseases affected by genetic polymorphisms, especially in countries with higher proportions of such polymorphisms. The current standard of applying global risk functions to local exposure data should be replaced by country-specific risk functions whenever possible. Country-specific risk functions should also be applied for other risk factors than alcohol.^1^^,^^51^ This will allow for better estimation of the burdens caused by risk factors and consequently better informed policy measures.
